# Exploring the relationships between composite scores of disease severity, seizure-freedom and quality of life in Dravet syndrome

**DOI:** 10.1186/s42466-022-00186-9

**Published:** 2022-06-06

**Authors:** Adam Strzelczyk, Gerhard Kurlemann, Thomas Bast, Ulrich Bettendorf, Gerhard Kluger, Thomas Mayer, Bernd A. Neubauer, Tilman Polster, Sarah von Spiczak, Regina Trollmann, Markus Wolff, Toby Toward, Jens Gruenert, Eddie Gibson, Clive Pritchard, Joe Carroll, Felix Rosenow, Susanne Schubert-Bast

**Affiliations:** 1 Epilepsy Center Frankfurt Rhine-Main and Department of Neurology, Goethe-University Frankfurt, Schleusenweg 2-16 (Haus 95), 60528 Frankfurt am Main, Germany; 2 Center for Personalized Translational Epilepsy Research (CePTER), Goethe-University Frankfurt, Frankfurt am Main, Germany; 3 Epilepsy Center Hessen and Department of Neurology, Philipps-University Marburg, Marburg (Lahn), Germany; 4 Department of Neuropediatrics, University of Münster, Münster, Germany; 5 Epilepsy Center Kork, Kehl-Kork, Germany; 6 Faculty of Medicine, University of Freiburg, Freiburg i. Br., Germany; 7 Neuropediatric Practice, Hirschaid, Germany; 8 Epilepsy Center for Children and Adolescents, Clinic for Neuropediatrics and Neurorehabilitation, Schön Klinik Vogtareuth, Vogtareuth, Germany; 9 Research Institute “Rehabilitation, Transition, and Palliation”, PMU Salzburg, Salzburg, Austria; 10 Epilepsy Center Kleinwachau, Radeberg, Dresden, Germany; 11 Department of Neuropediatrics, Justus-Liebig-University Giessen, Giessen, Germany; 12 Epilepsy Center Bethel, Bielefeld, Germany; 13 Northern German Epilepsy Center for Children and Adolescents, Raisdorf, Kiel, Germany; 14 Department of Neuropediatrics, Friedrich-Alexander University, Erlangen, Germany; 15 Department of Pediatric Neurology, Vivantes Klinikum Neukölln, Berlin, Germany; 16 Zogenix International Limited, Maidenhead, United Kingdom; 17 Wickenstones Ltd., Abingdon, United Kingdom; 18 Department of Neuropediatrics, Goethe-University Frankfurt, Frankfurt am Main, Germany

**Keywords:** Dravet syndrome, Composite endpoint, Statistical analysis, Seizures, Symptoms, Quality of life

## Abstract

**Background:**

In Dravet syndrome (DS), a rare epileptic and developmental encephalopathy, the effectiveness of a new treatment is predominantly measured in terms of seizure frequency. However, this may not fully capture the impact of a treatment on the broader aspects of the syndrome and patients’ health-related quality of life (HRQoL). Using a previously published survey which collected data from DS patients and their carers on the broader manifestations of their syndrome, their HRQoL, and their experience of seizures, this study created composite measures of symptom severity to offer new perspectives on the multifaceted aspects of this rare condition.

**Methods:**

Survey responses on the severity of physical and psychosocial symptoms were combined with independent assessments of disability and care need, to generate three composite symptom scores assessing the manifestations of DS (physical, psychosocial and care requirements). Variation in HRQoL was investigated in multiple regression analyses to assess the strength of association between each of these composite measures and three forms of seizure measures (seizure frequency, days with no seizures and longest interval without seizures), as experienced over a 4- and 12-week period.

**Results:**

Composite scores were calculated for a cohort of 75 primarily paediatric patients who were enrolled in the study. Strong associations were found between each of the three composite symptom scores and each of the three seizure measures, with the regression coefficient on symptom score highly significant (*p* ≤ 0.001) in all nine comparisons. Separate regressions using predictors of HRQoL (Kiddy KINDL and Kid KINDL) as the dependent variable were inconclusive, identifying only behavioural/attention problems and status epilepticus as significant predictors of HRQoL.

**Conclusions:**

These results allow the development of a composite score that may be useful in developing a clinical understanding of the severity of DS for an individual patient and establishing their treatment goals. Where measurement of long-term sequalae of disease is not feasible, such as clinical trials, correlation of the composite score with experience of seizures and seizure-free periods may allow a better contextualisation of the results of short-term assessments.

***Trial registration*:**

German Clinical Trials Register (DRKS), DRKS00011894. Registered 16 March 2017, http://www.drks.de/ DRKS00011894.

## Background

Dravet syndrome (DS) is an epileptic and developmental encephalopathy which, in addition to its high seizure burden, is characterised by a broad range of manifestations affecting patients’ health and wellbeing [3]. Although classified as a form of epilepsy, it is common for patients with DS to experience complex and progressive manifestations associated with developmental, social, cognitive and other impairment, alongside their frequent seizures [1].

Surveys of DS patients and their caregivers have found positive associations between improved health-related quality of life (HRQoL) and reduced frequency of seizures [6] and negative associations between the presence of manifestations such as motor impairments or behavioural difficulties and better HRQoL [2]. Concepts found by clinician ratings to be particularly important to the wider impact of DS on patients and carers include seizures, patients’ cognitive functioning, the caregiver’s daily activities and social functioning of the caregiver [9].

The main therapeutic goal for patients with DS is managing the frequency of seizures; an additional goal is to minimise other manifestations of the syndrome, including cognitive disabilities, behavioural difficulties and psychiatric problems [19, 23]. Due to the individualised and complex nature of DS, understanding and valuing the contribution of a new treatment for DS requires insight into patients’ subjective experience of treatment, alongside a broad understanding of the relationships between symptomatology, as well as their experience of seizures and HRQoL. In this study, we used an existing data set, collected amongst DS patients and their caregivers, combining cross-sectional data on physical and psychosocial disease manifestations with measures of resource utilisation captured by the German social system into composite symptom scores. Statistical analyses were then conducted exploring the relationships between the composite symptom scores and longitudinal data on seizure experience. This study examines the use of these scores in a largely paediatric cohort, but the methods could be further explored in DS patients of all ages in Germany.

## Methods

### Survey data

Analysis of the survey data on which the current research was based has been reported elsewhere [18, 20] [15]. The survey collected data until 2018 from paediatric and young adult patients with DS and their caregivers in regions throughout Germany. Data was collected using a retrospective questionnaire and a prospective 12-week diary.

In the retrospective questionnaire, caregivers answered questions on their child’s behalf about their disease, including comorbidities and HRQoL measures. Comorbidities measured were chronic infections, disturbance of motor skills and movement coordination, attention deficit disorder, delayed speech development, behavioural problems, muscular hypotension and cognitive disorders. This data was supplemented with information on the presence or absence of a disability (Grad der Behinderung) and the child’s care level (Pflegeversicherung), both provided by the caregiver but having been independently assessed. In Germany, a disability degree [21] score is assigned to patients by the social security office (Versorgungsamt) [5]. A care level is assigned to patients by the nursing care insurance system (Pflegeversicherung) that includes a mandatory medical assessment by an physician [13]. HRQoL in paediatric and adolescent patients was respectively assessed by the Kiddy KINDL in those aged 4–6 years old and by the Kid KINDL in those aged 7–17 years old [14]. Both instruments measured HRQoL based on physical, emotional, self-esteem, relationships with family, relationships with friends and schooling sub-domains.

In the 12-week-diary, caregivers chronicled their child’s day-to-day experiences with DS, including their daily seizure count. Caregivers recorded the number of seizures experienced by their child on a daily basis for 12 weeks, broken down by type of seizure [18].

The dataset used in the current analysis consisted of the 75 subjects who returned both the retrospective questionnaire and prospective diary.

### Seizure measures

Each patient was assigned three 4-week and three 12-week seizure measures using seizure data from the 12-week diary: number of (tonic–clonic) seizures (0–363), days in (tonic–clonic) seizure remission (13–84) and longest period in (tonic–clonic) seizure remission (2–84; defined as the maximum number of consecutive days without a tonic–clonic seizure in a 4- and 12-week period). Convulsive seizures were defined as tonic–clonic seizures for the purposes of this study. In addition, patients were asked whether they had ever experienced a status epilepticus.

The analysis focussed on tonic–clonic seizures as these were experienced by a greater proportion of subjects than any other single type of seizure and because the reported numbers were considered more reliable than for other types of seizure. Twelve-week seizure measures were considered more reliable indicators of seizure experience than the four-week equivalents. As the estimate of seizure-free interval could be influenced by the truncation of the data at the end of the 12-week diary period, this was the least preferred of the three seizure measures.

### HRQoL measures

Each patient was assigned an HRQoL measure, based on their caregivers’ responses collected by proxy on the Kiddy KINDL (for 4–6 year old children; 37.5–88.3) or Kid KINDL (for 7–17 year old children; 26.1–80.2) from the questionnaire.

### Composite symptom scores

Each patient was assigned a composite symptom score, which combine measures of physical and social impact, and measures of care requirement. The composite symptom scores were developed based on an existing dataset and based on the authors’ judgement rather than empirical consideration of the measurement properties associated with the individual scales or a formal process of consultation with experts in the disease area. Three variations of the composite symptom score were tested:

- Composite symptom score one (CS1): sum of the physical domain score and psychosocial domain score
- Composite symptom score two (CS2): sum of the physical domain score and psychosocial domain score and care requirement score
- Composite symptom score three (CS3): sum of the physical domain score and psychosocial domain score and care requirement score weighted to the same range of scores as the physical and psychosocial domain scores

In all domain and composite symptom scores, a higher score indicates more severe symptoms reported.

Together, the physical and psychosocial domains captured the comorbidities of the patient. The physical domain comprised scores for motor movement problems (MM), muscular hypotonia (MH) and muscular spasticity (MS). The psychosocial domain comprised scores for behaviour and attention problems (BA), language and speech problems (LS) and cognitive disorders (CD). MM, MH, MS, BA, LS, CD were based on questionnaire data (Table 1).

**Table 1:** Composite symptom scores and their components

Variable	Definition	Range of scores
*Composite symptom score*		
CS1	CS2 = MM + MH + MS + BA + LS + CD	0–18
CS2	CS2 = CS1 + D + CG	0–24
CS3	CS3 = CS1 + 1.5 x (D + CG)	0–24
*Domain*		
Physical	MM + MH + MS	0–9
Psychosocial	BA + LS + CD	0–9
Care requirement	D + CG	0–6
*Sub-domains*		
MM	0 = no problem, 1 = minor problem, 2 = moderate problem, 3 = severe problem	0–3
MH	0 = no problem, 1 = minor problem, 2 = moderate problem, 3 = severe problem	0–3
MS	0 = no problem, 1 = minor problem, 2 = moderate problem, 3 = severe problem	0–3
BA	0 = no problem, 1 = minor problem, 2 = moderate problem, 3 = severe problem	0–3
LS	0 = no problem, 1 = minor problem, 2 = moderate problem, 3 = severe problem	0–3
CD	0 = no problem, 1 = minor problem, 2 = moderate problem, 3 = severe problem	0–3
D	0 = no disability ID, 1 = disability score 10–30%, 2 = disability score < 50% and eligible for financial compensation, 3 = disability score ≥ 50%^1^	0–3
CG	0 = not in need of care (level 1)^2^; reimbursement received €689^3^, 1 = not in need of care (level 1)^2^; reimbursement received €1,298^3^, 2 = no care level but in need of care (level 2)^2^; reimbursement received €1,612^3^, 3 = receiving care (level 3)^2^; reimbursement received €1,995^3^	0–3

*MM* motor movement problems score; *MH* muscular hypotonia score; *MS* muscular spasticity score; *BA* behaviour and attention problems score; *LS* language and speech problems score; *CD* cognitive disorders score; *D* disability score; *CS* care grade score

^1^Described legally as a severe disability [21]

^2^based on the pre-2017 care level rating expressed on the 1–3 (Pflegebedürftigkeit—Need of care) scale [13]

^3^based on Die Pflegestärkungsgesetze I-III—Nursing improvement laws I-III, 2017 [7]

The care requirement domain comprised a disability score (D) and care grades score (CG) and a such, captured the patient’s resource use. D and SC were based on disability and care-level data collected by the questionnaire and re-categorised to a broad composite measure on a simple zero-to-six scale to which could be generalised to settings beyond the specific German context in which the measures were originally applied.

### Statistical analyses

To understand the relationship between the composite scores and seizure profile, linear (OLS) regressions were estimated using each of the composite scores as the independent variable and each of the continuous seizure measures as the dependent variable. Age and gender were added as control variables to pick up any variation which might be due purely to differences in patient profile.

In addition, multivariate regressions were carried out to help explain the variation in Kiddy KINDL and Kid KINDL in terms of comorbidities, seizure measures and patient demographic characteristics (control variables). A step-down regression procedure was implemented in which variables were iteratively removed until all variables remaining in the regression model were significant at the 10% significance level. As the Kid KINDL and Kiddy KINDL were developed and validated for separate age groups, statistical analysis conducted on HRQoL was conducted separately for each subsample.

### Software

The development of the composite symptom scores and all statistical analyses were conducted using STATA 15 [17]. Figures were created using Microsoft Excel [8].

## Results

### Seizures and disease severity

#### Domain and composite scores

The majority of patients had low to medium physical domain scores (93% had scores between 0 and 6 on a scale of 9) and medium to high care requirement scores (92% had scores between 3 and 6 on a scale of 6). Scores in the psychosocial domain were distributed equally across low (0–2), medium (4–6) and high (7–9) (Table 2).

**Table 2:** Distribution of physical, psychosocial, and care requirement domain scores in survey sample (*n* = 75)

Domain	Mean (SD)	Minimum	Maximum	Proportion of patients (%) reporting each score		
				Score 0–3	Score 4–6	Score 7–9
Physical	3.52 (2.10)	0	9	48%	45%	7%
Psychosocial	4.83 (2.62)	0	9	33%	33%	33%
				Score 0–2	Score 3–4	Score 5–6
Care requirements	4.35 (1.59)	0	6	8%	39%	53%

See Table 1 for definition of physical, psychosocial and care requirement domains

*SD* standard deviation

**Fig. 1:**
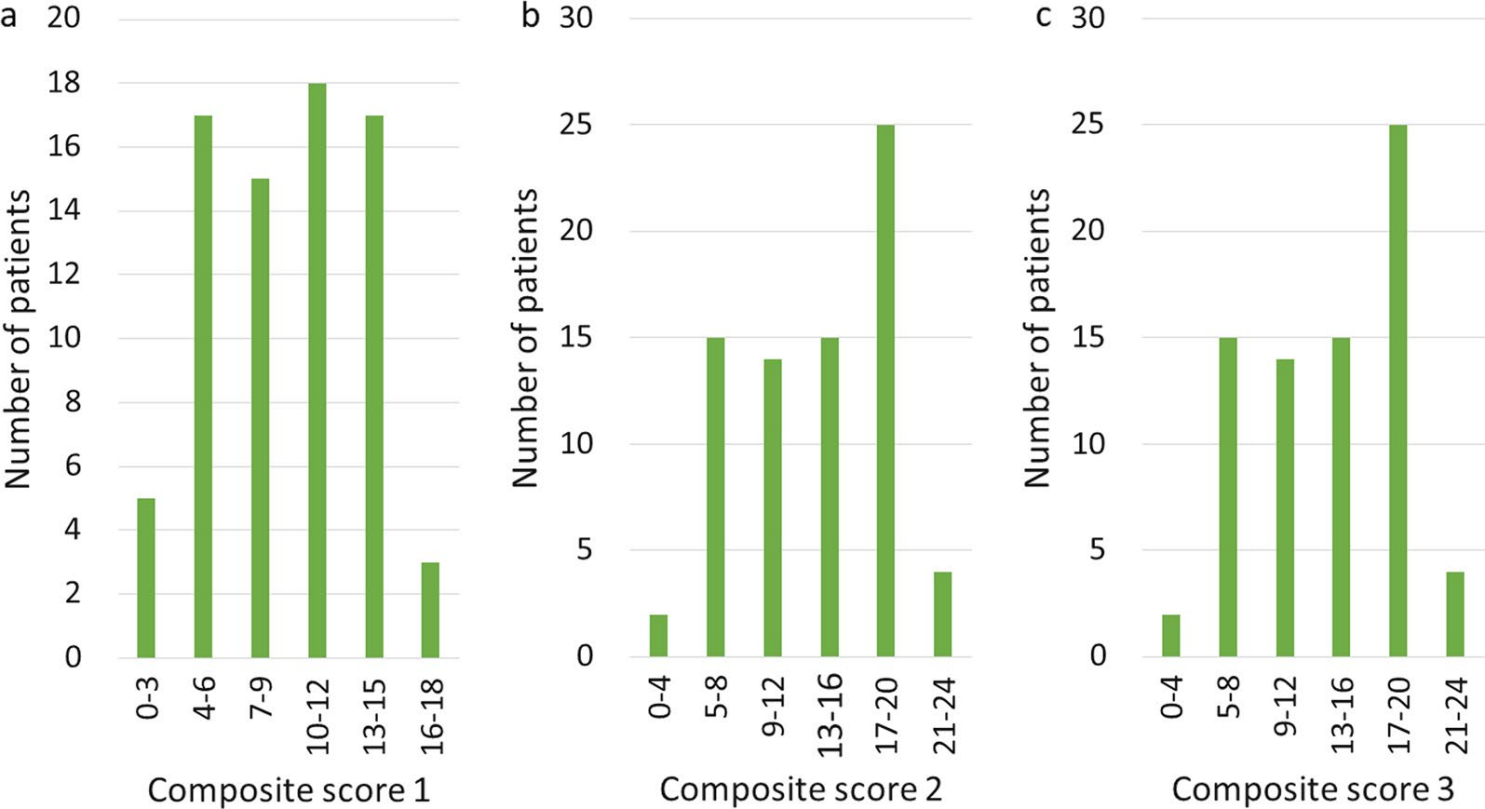
Distribution of composite scores in survey sample (*n* = 75). *Notes* See Table 1 for definition of composite scores 1, 2 and 3

The distribution of the three composite symptom scores showed few patients having the highest or lowest scores, with most patients scoring between 4–15 (CS1) and 5–20 (CS2 and CS3) (Fig. 1).

#### Regression analysis

The regressions of each composite symptom score against each of the seizure measures (seizure-free intervals, seizure-free days and total seizures) showed consistently strong associations. The coefficient on composite symptom score was highly significant (*p* ≤ 0.01) in all comparisons, a result which held true for both the twelve-week and four-week seizure measures. Table 3 presents the results for each composite score regressed on each tonic–clonic seizures measure. Neither of the two control variables, age and sex, was found to be significant (coefficients and P-values not reported here).

**Table 3:** Regression analysis of composite symptom scores on 12-week tonic–clonic seizure measures

Dependent variable: Longest seizure-free interval (12 weeks)		
Explanatory variable	Coefficient	*P* > *t*
CS1	− 3.24	< 0.01
CS2	− 2.71	< 0.01
CS3	− 2.38	< 0.01

Dependent variable: Seizure-free days (12 weeks)		
Explanatory variable	Coefficient	*P* > *t*
CS1	− 1.52	< 0.01
CS2	− 1.29	< 0.01
CS3	− 1.16	< 0.01

Dependent variable: Total seizures (12 weeks)		
Explanatory variable	Coefficient	*P* > *t*
CS1	4.57	0.01
CS2	3.91	< 0.01
CS3	3.51	0.01

See Table 1 for definition of CS 1, 2 and 3

*CS1* composite symptom score 1; *CS2* composite symptom score 2; *CS3* composite symptom score 3

**Fig. 2:**
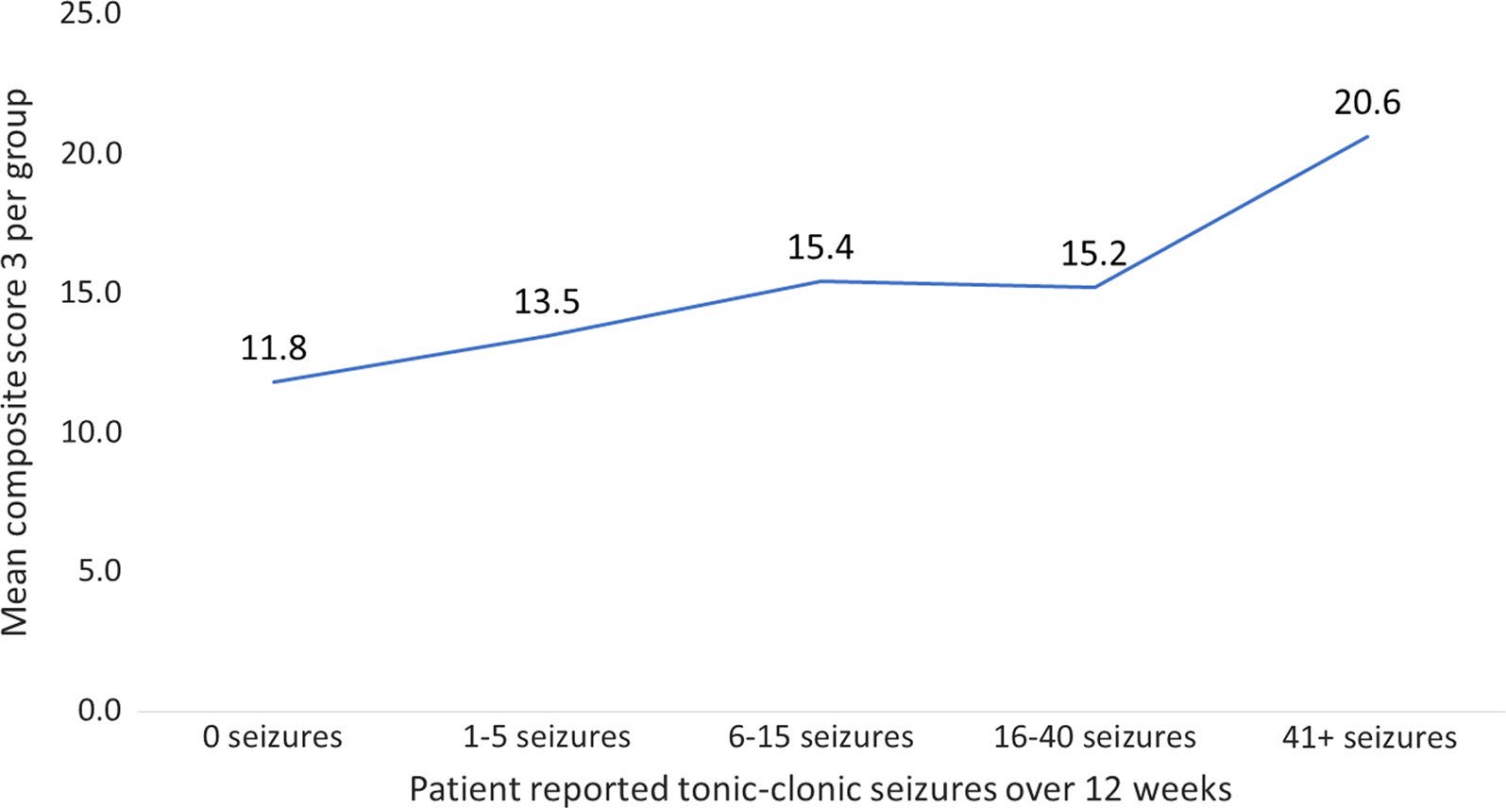
Mean composite symptom score three by seizure count. *Notes* See Table 1 for definition of CS3. Abbreviation: CS3, composite symptom score 3

The directions of the signs for the coefficients on composite scores were as expected. When using total number of seizures as the dependent variable, the coefficients on all the composite scores were positive. Those with higher disease severity (i.e. higher composite symptom scores) were more likely to experience more frequent tonic–clonic seizures, and vice versa. Where longest seizure-free interval and total seizure-free days were used as the dependent variables, the coefficients of the composite symptom scores were negative. Those with higher disease severity had shorter intervals between tonic–clonic seizures and fewer days without any tonic–clonic seizures, while those with composite symptom scores had longer intervals between tonic–clonic seizures and more days without any tonic–clonic seizures. Figure 2 illustrates the relationship between composite symptom scores and seizure measures by showing the upward trend in mean CS3 as the number of tonic–clonic seizures increases.

### The associations of seizures and other manifestations of DS with HRQoL

Using a step-down regression approach to iteratively eliminate those variables which did not have a significant coefficient in each successive regression equation, we obtained slightly different results between the two HRQoL measures. In the case of the younger age group (4–13 years old, *n* = 37; Kiddy KINDL), all seizure measures were removed from the regression by the step-down selection procedure and the final model included only behavioural/attention problems (*p* < 0.007) and status epilepticus (*p* < 0.005) as significant predictors of HRQoL (Kiddy KINDL) in this data set.

Within the older age group (14–17 years old, *n* = 20; Kid KINDL), speech and language problems were the only variable found to be statistically significant at the 10% cut-off (*p* = 0.06). As with the Kiddy KINDL, all seizure variables had been eliminated in the final model after implementation of the step-down selection procedure. Results of regression models estimated using the variables remaining at the conclusion of the step-down regression are reported in Table 4.

**Table 4:** Final HRQoL regression results following the step-down regression approach

Dependent variable: Kiddy KINDL			
Explanatory variables	Coefficient	*t*	*P* > *t*
BA	− 3.93	− 2.9	0.01
Previously experienced a status epilepticus	− 11.94	− 3.03	0.01

Dependent variable: Kid KINDL			
Explanatory variables	Coefficient	*t*	*P* > *t*
LS	4.49	2.05	0.06

The Kiddy KINDL is an HRQol instrument for children, the Kid KINDL for 7–17 for adolescents

*BA* behaviour and attention problems score; *HRQoL* health-related quality of life; *LS* language and speech problems score

## Discussion

### Analysis of the relationship between HRQoL, seizures, and other manifestations

This study represents the continuation of a body of literature in DS investigating the relationships between seizure experience, the broader manifestations of DS and patient HRQoL, as well as its impact on carers. Despite the documented associations between these three aspects of the disease, gaps remain in our understanding of the value to patients of treatments which reduce seizure counts. In particular, the evidence is inconclusive on the impact that a change in seizure metrics can have on broader measures of patient functioning and well-being for both patients and caregivers.

This is consistent with other research which has found several comorbidities to be associated with HRQoL, such as seizure control, behaviour, cognitive and motor problems [2].

In multivariate regression, we found behavioural and attention problems, speech and language problems, and status epilepticus to be associated with HRQoL. This is consistent with previous studies which have found behavioural problems to be a strong driver of HRQoL in DS patients [4, 12], with more recently published research finding behavioural problems to be the strongest predictor of HRQoL in DS [16]. Our finding that behavioural problems were significantly related to the Kiddy KINDL but not the Kid KINDL may, however, be symptomatic of the difficulties of interpreting regression results with the small sample sizes available, as the analysis was unable to detect a significant relationship for a number of other variables. Previous analysis of HRQoL in this sample found that children with DS scored comparatively poorly in the physical wellbeing, friends, and schooling domains as compared with patients with refractory epilepsy without encephalopathy [18, 20]. This may indicate that these domains are influenced by the comorbidities unique to DS which are not found in other epilepsies.

Although multivariate regression is the theoretically most robust approach to analysing variation in HRQoL, the restricted sample size is only one of several challenges with collecting and reporting HRQoL data in this context. A further significant consideration when attempting to measure and value treatment benefits in DS is the conceptual and practical difficulties in assessing HRQoL experienced by paediatrics and young adults, particularly at younger ages and when collected by proxy. In DS patients, who typically experience cognitive and communication difficulties, these issues are likely to be magnified.

### General disease severity and seizures

With these caveats in mind, the relationships between seizures and the broader manifestations of DS formed the focus of the analysis in this study. The significant relationships found between seizure measures and composite scores suggest that extended periods of seizure remission (whether expressed in terms of seizure frequency or periods free of seizures) are associated with overall better physical and psychosocial functioning in DS. Those who experienced the more severe seizure symptoms of DS were also likely to experience more severe non-seizure manifestations. This highlights the potential contribution that earlier and more effective use of interventions to prevent seizures may make, not only in reducing an acute hazard to health from injury or death, but also in its impact on the broader multidimensional aspects of DS [2]. Furthermore, although longer term evidence is needed, it may be hypothesised that early use of interventions that reduce seizure frequency and create longer intervals of time between seizures might alter the progressive course of DS.

DS is a complex disease with multiple clinical manifestations. Therefore, single symptoms, including seizure frequency, can provide information about individual aspects of the underlying disease, but the development of a true picture of disease severity relies on an understanding of all the clinical effects. The advantage of a composite score is that it can allow for heterogeneity in DS patients’ symptomatology [3] whereby the severity of the disease’s effects can vary between patients on the different individual manifestations of DS. While previous research has found the magnitude of cognitive and behavioural impairment of patients with DS to be related to seizure frequency [6], and the current study found a relationship between seizures and some broader manifestations of DS, this was not replicated across all measures. For example, we failed to find a consistent relationship between seizure measures and speech and language problems.

The possibility of extrapolating a patient’s disease characteristics from their experience of convulsive (in particular tonic–clonic) seizures suggests a means of exploring the connection between a treatment’s impact on seizures and HRQoL. Clinical trials may be able to demonstrate short-term effects in terms of seizure reduction but may lack the sample size or the duration of follow-up to assess the broader and mid to longer-term impacts of treatment. The options for collecting significant amounts of data using wide-ranging surveys with multiple measures of disease impact are generally not available when choosing outcome measures for use in the trial setting. In this context, it is potentially useful to explore summary measures which are able to encompass the global burden of disease.

This study resonates with the search by Nabbout et al. [9] for a limited set of clinically meaningful endpoints which can potentially be measured and detect change over time with treatment in DS. In addition to seizures, they propose patient cognitive functioning (expressive and receptive communication) alongside caregiver daily activities and caregiver social functioning as measures which could usefully be incorporated into clinical trials [11] [10]. In the future, it is envisaged that the chosen endpoints could be collapsed into a single endpoint for use in clinical trials [9]. The research presented in this study complements that of Nabbout et al. by suggesting that it may be possible to use evidence on seizure experience as a proxy for a broad set of impacts which can be summarised on a single scale. The ideas presented here may be extended to develop composite symptom measures in other developmental and epileptic encephalopathies, such as Lennox-Gastaut syndrome and tuberous sclerosis complex (TSC). Recent cross-sectional studies of TSC patients in Germany may provide a data source for further research in this area [22, 24]. In addition, further research is needed for all developmental and epileptic encephalopathies to understand the effect of seizure limiting anti-epileptic drugs on non-seizure composite scores.

### Limitations of the analysis

The main limitation of the current study in attempting to explain variations in HRQoL was, as already noted, the small number of subjects available. The matching of questionnaire and diary samples reduced the overall sample from 93 to 75 while restricting further to children and young people limited the number to 57 who completed either the Kiddy KINDL assessed in 4–6 year olds (37 respondents) or Kid KINDL assessed in 7–17-year olds (20 respondents). The separate regressions estimated in each group therefore need to be interpreted with caution.

Secondly, where data on HRQoL was limited by sample size, prospective diary data on seizure experience was limited by the time horizon over which it was collected. As seizure data was restricted to twelve weeks, this resulted in some truncation in the seizure distributions, with a sizeable proportion of the sample reporting few to no seizures in the twelve-week period. This could result in the regressions underestimating the significance of the relationship between seizure measures and other variables and limits the generalisability of the results to those patients who experience more infrequent seizures. However, it does constrain the length of time between responses to the questionnaire and the diary, giving reassurance that the data collected in each was consistent. Further longitudinal research recording other disease manifestations in addition to seizure experience over longer time horizons is needed to extend the conclusions of these analyses to those DS patients experiencing less frequent seizures.

## Conclusions

This research has used the re-analysis of a real-world observational dataset combining retrospective caregiver assessments of the way in which DS manifests itself in a sample of children and young people, together with prospective diary data on seizure experience, to explore the links between seizures, the broader manifestations of DS and patient HRQoL.

Composite measures constructed from a combination of individual comorbidity measures were found to have consistently significant associations with seizure measures and so may play an important part in the scoring of patients to establish their clinical requirements and treatment goals. In the clinical setting, we propose that a summary measure which captures a wide range of disease manifestations can be used to classify patients for the purposes of assessing their holistic disease burden. A simple additive combination of caregiver-assessed comorbidity measures together with weighted care level and disability scores, such as in our CS3, can provide useful additional information about the overall health and well-being of DS patients. Although disability and care need, in the current data set, are assessed by independent administrative bodies, the simplified 0 to 3 scaling is potentially transferrable to a range of other settings. Real-world evidence on the distribution of patients between care or disability categories such as those used here could also provide indicative benchmarks for categorising other patient groups.

Correlation of the composite score with seizure experience might be used to capture a more rounded view of the value of seizure reduction in DS in settings, such as clinical trials, where broad assessments may be infeasible or studies are of insufficient duration to record longer-term changes in patient health. In addition, this approach may help to address some of the conceptual and measurement issues in assessing HRQoL in groups such as children and young people with DS. To our knowledge this is the first study to quantify a patient-focused measure of the non-seizure manifestations of DS for statistical analysis against measures of seizures and seizure freedom. Further research is needed to investigate the issues it raises, such as the importance of seizure freedom independently of seizure counts, and to validate its approach towards understanding the subjective experience associated with the complex array of impacts comprising DS.

## Data Availability

The datasets used and/or analysed during the current study are available from the corresponding author on reasonable request.

## Abbreviations

BoI: Burden of illness
DS: Dravet syndrome
GdB: Grad der Behinderung
HRQoL: Health-related quality of life
OLS: Ordinary least-squares
SUDEP: Sudden unexplained death in epilepsy

## References

[1] Anwar A, Saleem S, Patel UK, Arumaithurai K, Malik P (2019). Dravet syndrome: An overview. Cureus.

[2] Brunklaus A, Dorris L, Zuberi SM (2011). Comorbidities and predictors of health-related quality of life in Dravet syndrome. Epilepsia.

[3] Dravet C (2011). Dravet syndrome history. Developmental Medicine and Child Neurology.

[4] Dravet C, Bureau M, Oguni H, Fukuyama Y, Cokar O (2005). Severe myoclonic epilepsy in infancy: Dravet syndrome. Advances in Neurology.

[5] Familienratgeber.de. (2020). Versorgungsamt. Retrieved from https://www.familienratgeber.de/schwerbehinderung/schwerbehindertenausweis/versorgungsamt.php

[6] Lagae L, Brambilla I, Mingorance A, Gibson E, Battersby A (2018). Quality of life and comorbidities associated with Dravet syndrome severity: A multinational cohort survey. Developmental Medicine and Child Neurology.

[7] Lötzerich, U. (2018) Die Pflegestärkungsgesetze (PSG) I, II und III. Retrieved from https://www.pflege.de/pflegekasse-pflegefinanzierung/pflegeleistungen/pflegegeld/

[8] Microsoft. (2018). Microsoft Excel. https://office.microsoft.com/excel: Microsoft Corporation.

[9] Nabbout R, Auvin S, Chiron C, Irwin J, Mistry A, Bonner N, Williamson N, Bennett B (2018). Development and content validation of a preliminary core set of patient- and caregiver-relevant outcomes for inclusion in a potential composite endpoint for Dravet syndrome. Epilepsy & Behavior.

[10] Nabbout R, Auvin S, Chiron C, Thiele E, Cross H, Scheffer IE, Schneider AL, Guerrini R, Irwin J, Mistry A, Grimes R, Bennett B, Williamson N (2019). Perception of impact of Dravet syndrome on children and caregivers in multiple countries: Looking beyond seizures. Developmental Medicine and Child Neurology.

[11] Nabbout R, Dirani M, Teng T, Bianic F, Martin M, Holland R, Chemaly N, Coque N (2020). Impact of childhood Dravet syndrome on care givers of patients with DS, a major impact on mothers. Epilepsy & Behavior.

[12] Nolan K, Camfield CS, Camfield PR (2008). Coping with a child with Dravet syndrome: Insights from families. Journal of Child Neurology.

[13] Pflege.de. (2020). Pflegekasse und Pflegerecht. Retrieved from https://www.pflege.de/pflegekasse-pflegerecht/

[14] Ravens-Sieberer U, Ellert U, Erhart M (2007). Gesundheitsbezogene lebensqualität von kindern und jugendlichen in deutschland. Bundesgesundheitsblatt–Gesundheitsforschung–Gesundheitsschutz.

[15] Schubert-Bast S, Wolff M, Wiemer-Kruel A, Spiczakvon S, Trollmann R, Reif PS, Pritchard C, Polster T, Neubauer BA, Mayer T, Macdonald D, Kurlemann G, Kluger G, Klein KM, Kieslich M, Irwin J, Herting A, Carroll J, Bettendorf U, Bast T, Rosenow F, Strzelczyk A (2019). ) Seizure management and prescription patterns of anticonvulsants in Dravet syndrome: A multicenter cohort study from Germany and review of literature. Epilepsy & Behaviour.

[16] Sinoo C, de Lange IM-L, Westers P, Gunning WB, Jongmans MJ, Brilstra EH (2019). Behavior problems and health-related quality of life in Dravet syndrome. Epilepsy & Behavior.

[17] StataCorp. (2017). Stata: release 15, statistical software.

[18] Strzelczyk A, Kalski M, Bast T, Wiemer-Kruel A, Bettendorf U, Kay L, Kieslich M, Kluger G, Kurlemann G, Mayer T, Neubauer BA, Polster T, Herting A, von Spiczak S, Trollmann R, Wolff M, Irwin J, Carroll J, Macdonald D, Pritchard C, Klein KM, Rosenow F, Schubert-Bast S (2019). Burden-of-illness and cost-driving factors in Dravet syndrome patients and carers: A prospective, multicenter study from Germany. European Journal of Paediatric Neurology.

[19] Strzelczyk A, Schubert-Bast S (2020). Therapeutic advances in Dravet syndrome: A targeted literature review. Expert Review of Neurotherapeutics.

[20] Strzelczyk A, Schubert-Bast S, Bast T, Bettendorf U, Fiedler B, Hamer HM, Herting A, Kalski M, Kay L, Kieslich M, Martin Klein K, Kluger G, Kurlemann G, Mayer T, Neubauer BA, Polster T, von Spiczak S, Stephani U, Trollmann R, Wiemer‐Kruel A, Wolff M, Irwin J, Carroll J, Pritchard C, Rosenow F (2019). A multicenter, matched case-control analysis comparing burden-of-illness in Dravet syndrome to refractory epilepsy and seizure remission in patients and caregivers in Germany. Epilepsia.

[21] Versorgungsmedizin-Verordnung, (2015). Retrieved from https://www.bmas.de

[22] Willems LM, Schubert-Bast S, Grau J, Hertzberg C, Kurlemann G, Wiemer-Kruel A, Bast T, Bertsche A, Bettendorf U, Fiedler B, Hahn A, Hartmann H, Hornemann F, Immisch I, Jacobs J, Kieslich M, Klein KM, Klotz KA, Kluger G, Knuf M, Mayer T, Marquard K, Meyer S, Muhle H, Müller-Schlüter K, Noda AH, Ruf S, Sauter M, Schlump JU, Syrbe S, Thiels C, Trollmann R, Wilken B, Zöllner JP, Rosenow F, Strzelczyk A (2021). Health-related quality of life in children and adolescents with tuberous sclerosis complex and their caregivers: A multicentre cohort study from Germany. European Journal of Paediatric Neurology.

[23] Wirrell EC (2016). Treatment of Dravet Syndrome. Canadian Journal of Neurological Sciences.

[24] Zöllner JP, Conradi N, Sauter M, Knuf M, Knake S, Kurlemann G, Mayer T, Hertzberg C, Bertsche A, Immisch I, Klein KM, Marquard K, Meyer S, Noda AH, von Podewils F, Schäfer H, Thiels C, Zukunft B, Schubert-Bast S, Grau J, Willems LM, Rosenow F, Reese JP, Strzelczyk A (2021). Quality of life and its predictors in adults with tuberous sclerosis complex (TSC): a multicentre cohort study from Germany. Neurological Research and Practice.

